# Follow‐up of intraocular retinoblastoma through the quantitative analysis of conserved nuclear DNA sequences in aqueous humor from patients

**DOI:** 10.1002/cjp2.296

**Published:** 2022-09-23

**Authors:** Maria Cuadrado‐Vilanova, Victor Burgueño, Leire Balaguer‐Lluna, Rosario Aschero, Helena Castillo‐Ecija, Jing Liu, Sara Perez‐Jaume, Guillem Pascual‐Pasto, Nagore G Olaciregui, Soledad Gomez‐Gonzalez, Genoveva Correa, Mariona Suñol, Paula Schaiquevich, François Radvanyi, Cinzia Lavarino, Jaume Mora, Jaume Catala‐Mora, Guillermo L Chantada, Angel M Carcaboso

**Affiliations:** ^1^ Institut de Recerca Sant Joan de Deu Barcelona Spain; ^2^ SJD Pediatric Cancer Center Barcelona Hospital Sant Joan de Deu Barcelona Spain; ^3^ Institut Curie CNRS, UMR144, SIREDO Oncology Center Paris France; ^4^ Institut Curie PSL Research University Paris France; ^5^ Pediatrics Hospital HM Nens Barcelona Spain; ^6^ Pathology Hospital Sant Joan de Deu Barcelona Spain; ^7^ Hospital de Pediatria JP Garrahan Buenos Aires Argentina; ^8^ CONICET Buenos Aires Argentina; ^9^ Ophthalmology Hospital Sant Joan de Deu Barcelona Spain

**Keywords:** eye, neoplasia, retinoblastoma, liquid biopsy, aqueous humor, droplet digital polymerase chain reaction, nuclear DNA, mitochondrial DNA, intravitreal chemotherapy, biomarkers

## Abstract

Fundoscopy is the standard method for diagnosis and follow‐up of intraocular retinoblastoma, but it is sometimes insufficient to discern whether tumors are inactivated following treatments. In this work, we hypothesized that the amount of conserved nuclear DNA sequences in the cell‐free DNA (cfDNA) fraction of the aqueous humor (AH) might complement fundoscopy for retinoblastoma follow‐up. To address our hypothesis, we developed highly sensitive droplet digital polymerase chain reaction (ddPCR) methods to quantify highly conserved DNA sequences of nucleus‐encoded genes (*GAPDH* and *B4GALNT1)* and of a mitochondrial gene, *MT‐ATP6*. We obtained AH samples during intravitreal treatments. We analyzed 42 AH samples from 25 patients with intraocular retinoblastoma and 11 AH from controls (non‐cancer patients). According to clinical criteria, we grouped patients as having progression‐free or progressive retinoblastoma. cfDNA concentration in the AH was similar in both retinoblastoma groups. Copy counts for nucleus‐derived sequences of *GAPDH* and *B4GALNT1* were significantly higher in the AH from patients with progressive disease, compared to the AH from progression‐free patients and control non‐cancer patients. The presence of mitochondrial DNA in the AH explained that both retinoblastoma groups had similar cfDNA concentration in AH. The optimal cut‐off point for discriminating between progressive and progression‐free retinoblastomas was 108 *GAPDH* copies per reaction. Among patients having serial AH samples analyzed during their intravitreal chemotherapy, *GAPDH* copies were high and decreased below the cut‐off point in those patients responding to chemotherapy. In contrast, one non‐responder patient remained with values above the cut‐off during follow‐up, until enucleation. We conclude that the measurement of conserved nuclear gene sequences in AH allows follow‐up of intraocular retinoblastoma during intravitreal treatment. The method is applicable to all patients and could be relevant for those in which fundoscopy evaluation is inconclusive.

## Introduction

Fundoscopy is the standard method to diagnose and follow‐up intraocular retinoblastoma, the tumor of the retina occurring in approximately 1 in 18,000 young children [1]. This clinical evaluation technique, however, is sometimes insufficient for the differential diagnosis of some retinoblastomas and it is not always sufficiently sensitive to discern whether intraocular tumors remain active or not following treatments. In this context, sensitive and specific laboratory techniques detecting viable tumors in biological samples might help address the unmet needs of fundoscopy in retinoblastoma diagnosis and follow‐up. Because the risk of tumor dissemination precludes biopsy at the intraocular location, such laboratory techniques must rely on samples obtained by minimally invasive methods such as fine needle aspiration of the anterior chamber [2]. Aqueous humor (AH) samples obtained through this process contain cell‐free DNA (cfDNA), partially from tumor origin, thus holding patient‐specific genomic alterations [3]. Because each tumor contains different genomic aberrations, namely mutations in the *RB1* gene, but also mutations in *BCOR* [4], *MYCN* amplifications [5], and larger somatic chromosomal alterations such as chromosome 6p gains [6], analysis of AH has been proposed as a tool for identifying the disease in a patient‐specific way [7]. Ultra‐low pass genome sequencing of cfDNA in AH can estimate the fraction of cfDNA derived from tumor cells in AH samples. This method has been used to follow‐up therapeutic response of retinoblastoma patients with at least three sequential AH samples obtained during chemotherapy [8]. Patient‐specific aberrations in genomic sequences of retinoblastoma are also detectable in human blood cfDNA using in‐depth sequencing with 20,000× raw coverage [9]. However, developing patient‐specific deep sequencing techniques is not feasible without a vast investment of resources, and a putative universal particularity of the cfDNA found in all retinoblastomas might thus be a more suitable biomarker for general use.

The composition of cfDNA in AH from retinoblastoma patients remains incompletely characterized, especially concerning the proportion of mitochondrial and nuclear DNA. A single normal cell contains only two copies of a nuclear DNA in the nucleus, whereas there are thousands of copies of mitochondrial DNA [10]. In some types of cancer, such as colorectal and papillary thyroid carcinomas, the proportion of mitochondrial and nuclear DNA circulating in plasma samples is different compared to that of patients without cancer [11, 12]. The mitochondrial‐to‐nuclear DNA ratio in the blood of healthy individuals is up to four times higher than in cancer patients [11, 12].

In this work, we hypothesized that AH samples obtained from patients with intraocular retinoblastomas would contain different amount of conserved nuclear DNA sequences in their cfDNA, depending on the load of viable tumor, proliferative status of the cancer cells, or response to treatment of each patient. To count DNA copies and to calculate the mitochondrial‐to‐nuclear DNA proportion in AH samples, we developed droplet digital polymerase chain reaction (ddPCR) techniques for specific sequences contained in the nucleus‐encoded genes *B4GALNT1* and *GAPDH* and in the mitochondrial gene *MT‐ATP6*. The sequences of *B4GALNT1* and *GAPDH*, both in chromosome 12, are highly conserved in retinoblastomas, and in genomic regions away from areas that include frequent alterations such as *RB1* (chromosome 13q) and *MYCN* (chromosome 2p) [4, 13]. In addition, the gene *B4GALNT1* produces the ganglioside GD2, which is highly expressed in retinoblastoma [14, 15]. In the present study, we detected patterns of change in the mitochondrial and nuclear DNA composition through the analysis of serial AH samples obtained during intravitreal treatments of responder and non‐responder patients.

## Materials and methods

### Collection of supernatants from primary cell cultures

The institutional review boards at Hospital Sant Joan de Deu (SJD, Barcelona, Spain; protocol M‐1608‐C) and Hospital JP Garrahan (HPG, Buenos Aires, Argentina; protocols 838 and 904) approved the collection of tumor tissues to establish primary cultures, under informed consent. As sources of cfDNA to set up the analytical methods, we used supernatants of nine primary retinoblastoma cultures established from the tumors of eight patients. We cultured cells in supplemented retinoblastoma medium (serum‐free medium with added growth factors), as described previously [16]. All cultures were under passage 20. As a quality control, we determined the short tandem repeat profiles of the primary cells, which we reported elsewhere [17].

To produce culture supernatants, we plated primary cells in 2 ml of supplemented retinoblastoma medium at a cell density of 4 × 10^6^ cells/well in six‐well plates. After 48 h, we collected supernatants and cell pellets by centrifugation at 400 × *g* for 4 min. We stored samples at −20 °C until cfDNA extraction.

### Extraction of cfDNA and genomic cell DNA

We isolated cfDNA from cell supernatants using QIAamp DNA Micro Kits (Qiagen, Hilden, Germany). To isolate genomic DNA from cell pellets, we lysed cells with Cell Lysis solution (158908, Qiagen) and precipitated the protein fraction with Protein Precipitation Solution (158912, Qiagen). Then, we precipitated the DNA with isopropanol and ethanol (Sigma Aldrich, Saint Louis, MO, USA) and rehydrated it with DNA Hydration Solution (158914, Qiagen). We quantified DNA using a NanoDrop 1000 Spectrophotometer (ThermoFisher Scientific, Waltham, MA, USA).

### Development of a ddPCR method to quantify *B4GALNT1* and *GAPDH* in cfDNA

To amplify nucleus‐encoded DNA gene sequences, we used primers for *B4GALNT1* (dHsaCPE5044772, Bio‐Rad, Hercules, CA, USA) and *GAPDH* (dHsaCPE5031597, Bio‐Rad). For mitochondrial DNA sequences, we used primers for *MT‐ATP6* (dHsaCPE5192289, Bio‐Rad). All primers were designed without intron‐spanning, to facilitate the amplification of genomic DNA sequences. To prepare samples for droplet generation we used Supermix for probes (no dUTP) (Bio‐Rad). We generated droplets using the QX200 ddPCR System (Bio‐Rad). We analyzed positive droplets using QuantaSoft Software (Bio‐Rad). We established the lower limit of detection (LOD) and the lower limit of quantification (LOQ) of the technique in three independent (inter‐assay) calibration experiments using genomic DNA obtained from the retinoblastoma primary cell culture HSJD‐RBT‐7 [15]. We selected these cells because they conserve an intact chromosome 12 and expression of *B4GALNT1* [15]. In each experiment, we quantified in triplicates (intra‐assay) gene copies of *B4GALNT* and *GAPDH* in serial 1:2 dilutions, and copies of *MT‐ATP6* in serial 1:2.7 dilutions. The greatest amount of DNA loaded in the 20 μl ddPCRs was 5.50 ng for nucleus‐encoded DNA analyses, and 0.2 ng for mitochondrial DNA. As negative controls, in each assay we ran blank samples in which we replaced the DNA fraction with injectable water. We defined the LOD as the lowest number of gene copies per reaction detected with at least three positive droplets in all the triplicates in the three independent calibration experiments. We defined the LOQ as the lowest number of gene copies per reaction with an inter‐assay coefficient of variation <20%. To evaluate the linearity of the technique, we calculated the coefficient of determination (*R* ^2^). We considered linearity acceptable when the slope for both analyzed genes was statistically different from zero and *R* ^2^ > 0.9.

To quantify nucleus‐encoded DNA gene sequences in culture supernatants and cell pellets we used 5 ng DNA in the ddPCRs. To quantify the mitochondrial DNA gene sequence, we used 0.2 ng DNA. We expressed the results as the number of copies per ng DNA.

### Collection and analysis of AH samples and *RetCam* images

We collected AH samples (100 μl volume) from retinoblastoma patients undergoing intravitreal chemotherapy from March 2018 to April 2022. The institutional review board at Hospital SJD approved the collection of samples from oncology patients (informed consent M‐1608‐C). This sampling procedure is associated with the withdrawal of AH to diminish the intraocular pressure before intravitreal treatments. On the same day of the AH sampling, we obtained representative fundoscopy images of the tumors using the *RetCam* (Clarity Medical Systems, Inc., Pleasanton, CA, USA). At the first AH collection of each patient, we obtained fundoscopy images and classified tumor chemotherapy response according to the Retinoblastoma Response Evaluation Criteria in Solid Tumors (RB‐RECIST) criteria [18]. In brief, the RB‐RECIST criteria define complete response (CR) for tumors and seeds when they disappear or when they show clinical stability for more than 6 months after cessation of local consolidation therapy. Partial response (PR) for tumors is defined as a decrease in apical tumor height by more than 30%, or clinical stability for less than 6 months, and for vitreous seeds PR is achieved when the density or number of seeds decreases unequivocally, or remains stable for less than 6 months. Stable disease (SD) for tumors is defined as a decrease in apical tumor height less than 30%, and for vitreous seeds as neither improvement nor progression. Progressive disease (PD) for tumors is defined as appearance of new lesions or an increase in tumor size by more than 30%, and for vitreous seeds PD is defined as increased number or density of the seeds.

Because our study included HA from patients with or without previous treatment, to perform the final analyses of AH as a marker of progressive disease we categorized retinoblastoma patients into two main groups. The first group, named ‘Progression‐free’, was composed of patients with clinically stable tumors or tumors responding to treatment, i.e. patients presenting with CR, PR, and SD according to RB‐RECIST criteria. The second group, named ‘Progressive RB’, was composed of patients with highly proliferative tumors, i.e. patients presenting with PD according to RB‐RECIST criteria and patients at initial diagnosis. A third group, named ‘Controls’, was composed of non‐retinoblastoma patients from whom we obtained AH during surgeries to treat other eye conditions, mostly cataracts.

We stored all AH samples at −20 °C. On the day of the analysis, we processed 20 μl AH for cfDNA extraction, as described previously for cell supernatants, and subsequently for ddPCR. To quantify nuclear and mitochondrial DNA genes we used 5 ng DNA in the ddPCRs. We expressed the results as the number of gene copies per reaction.

### Histopathology analysis of enucleated eyes

We analyzed tumors of enucleated patients included in the study. We used 4‐μm serial sections of formalin‐fixed paraffin‐embedded tumor samples and performed H&E staining and immunohistochemistry for detection of synaptophysin as a neuroendocrine marker, using a primary mouse monoclonal antibody SYNAP‐299‐L‐CE (PA0299, Leica Biosystems, Newcastle, UK). We performed automatic staining using a Leica Bond‐MAX autostainer (Leica Biosystems).

### Statistics

We performed linear regressions and statistical analyses using Graphpad v7.04 software (GraphPad Inc., San Diego, CA, USA). We represented aggregate data as median ± standard deviation. To compare two groups, we applied the Mann–Whitney test. To compare more than two groups, we used the Kruskal–Wallis test with Dunn's multiple comparison correction. We considered *p* < 0.05 as the threshold for significance.

To evaluate the sensitivity and specificity of the proposed methods to differentiate Progression‐free and Progressive RB groups, we built the receiver operating characteristic (ROC) curves using R v4.1.3 software (R Foundation for Statistical Computing, Vienna, Austria). To select an optimal threshold value (cut‐off point) for each marker we used the Youden index maximization method [19, 20]. We used Delong's method to compute the 95% confidence intervals (CIs) for the area under the curves.

## Results

### LOD and LOQ of selected nuclear and mitochondrial DNA genes, linearity of the techniques, and analysis of retinoblastoma cultures by ddPCR

**Figure 1:**
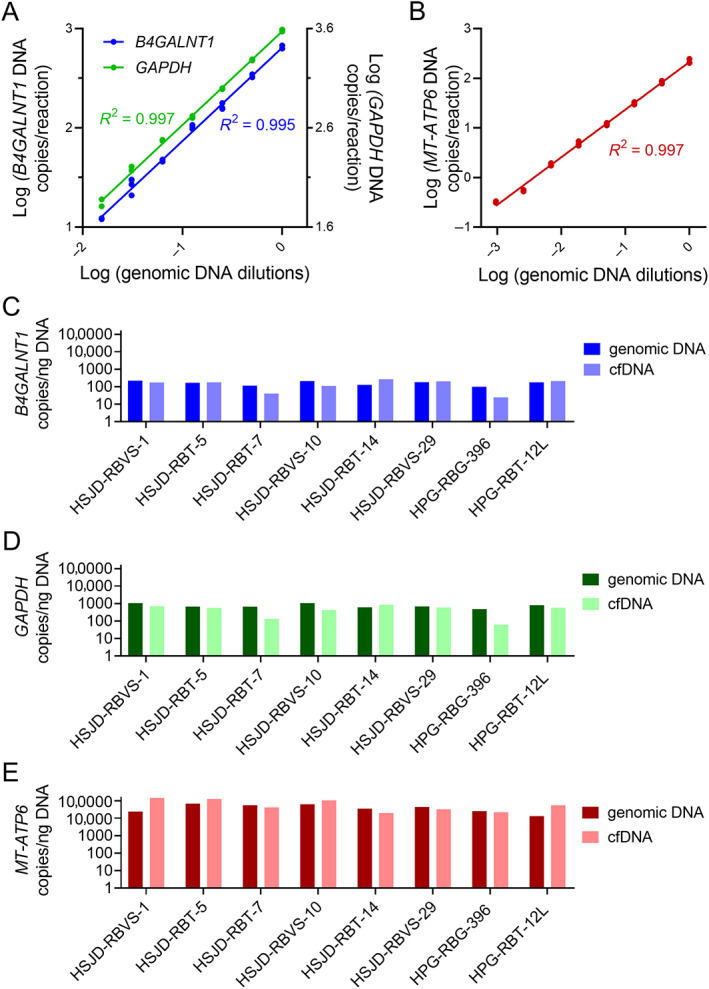
Linearity of the ddPCR technique and quantification of nuclear and mitochondrial DNA sequences in cell cultures. (A) Number of copies/ng DNA of *B4GALNT1* and *GAPDH* in serial dilutions (range 1–1:64) of genomic DNA of retinoblastoma. Genomic DNA input was 5.5 ng in dilution 1. Dots are intra‐assay means of each of the three independent calibration experiments. Regression curves for *B4GALNT1* and *GAPDH* follow a simple linear regression model. (B) Number of copies/ng DNA of *MT‐ATP6* in serial dilutions (range 1–1:369) of genomic DNA of retinoblastoma. Genomic DNA input was 0.2 ng in dilution 1. Dots are intra‐assay means of each of the three independent calibration experiments. Regression curve follows a simple linear regression model. (C) *B4GALNT1* copies in genomic DNA and supernatant cfDNA of primary retinoblastoma cultures. (D) *GAPDH* copies in genomic DNA and supernatant cfDNA of primary retinoblastoma cultures. (E) *MT‐ATP6* copies in genomic DNA and supernatant cfDNA of primary retinoblastoma cultures.

LOD and LOQ for nuclear‐encoded DNA sequences of *B4GALNT1* and *GAPDH* were 12 and 72 copies per ddPCR, respectively, corresponding to the dilution 1:64 of the initial input of 5.50 ng DNA. Mean inter‐assay *R* ^2^ was 0.995 for *B4GALNT1* and 0.997 for *GAPDH* in serial 1:2 dilutions of genomic DNA (Figure 1A). Slopes for both sequences were different from zero (*p* < 0.0001). We did not detect false positives in any of the analyzed blank samples. LOD and LOQ for mitochondrial DNA sequence of *MT‐ATP6* were 6.4 copies per ddPCR, corresponding to the dilution 1:369 of the initial input of 0.2 ng DNA. Mean inter‐assay *R* ^2^ was 0.997 (Figure 1B). The slope was different from zero (*p* < 0.0001).

All retinoblastomas released cfDNA to the culture supernatant, corresponding to 2.96% (0.70–6.32) of the genomic DNA (median and range of eight cell cultures; supplementary material, Table S1). Nuclear sequences were equally represented in the cfDNA and the genomic DNA. Specifically, median *B4GALNT1* copies were 174 (24–264) copies/ng cfDNA and 171 (98–221) copies/ng genomic DNA (Figure 1C). *GAPDH* copies were 568 (62–866) copies/ng cfDNA and 671 (481–1059) copies/ng genomic DNA (Figure 1D). The mitochondrial gene *MT‐ATP6* was abundant in the cfDNA (47,874; 19,877–143,046 copies/ng) and in the genomic DNA (39,090; 13,102–67,549 copies/ng) (Figure 1E). Because the ddPCR technique was more sensitive for *GAPDH* in retinoblastoma samples, we selected *GAPDH* as the nucleus‐encoded sequence to calculate the mitochondrial‐to‐nuclear DNA ratios in AH samples.

### Patient samples

We analyzed 42 AH samples from 25 retinoblastoma patients (clinical details in Table 1) and 11 AH from Controls (supplementary material, Table S2). Median age at retinoblastoma diagnosis was 7.6 months (range 1.0–35). At the time of the first AH sampling, 11 patients were included in the group Progression‐free (5 SD, 5 PR, and 1 CR) (Table 1). Patients 13, 15, and 21, who were included in this group, had a second AH sample analyzed, obtained after achieving CR. The remaining 14 patients, included in the group Progressive RB, presented very active and proliferating tumors, 12 of them with PD after treatment, and 2 with newly diagnosed treatment‐naïve retinoblastoma (Table 1). Patients 1, 2, 16, and 38 presented anterior chamber infiltration and were treated with 10–20 μg of intracameral topotecan following recommended guidelines [1] (Table 1). From patients 8, 16, and 20, we obtained serial AH (4, 8, and 5 samples, respectively) corresponding to different stages of their treatment. Four of the 25 patients were finally enucleated (Table 1).

**Table 1:** Clinical details of retinoblastoma patients

Patient ID	Age at diagnosis (month)	Treatments before AH sampling	Progression status and RB‐RECIST criteria at the time of AH sampling	Anterior chamber infiltration at the time of AH sampling	Enucleation	Reason for enucleation
1	1.4	SCT, IVC	Progressive (PD)	Yes	No	
2	1.0	SCT	Progressive (PD)	Yes	No	
3	2.2	IAC	Progressive (PD)	No	No	
4	4.0	SCT	Progression‐free (CR)	No	No	
5	8.0	SCT	Progressive (PD)	No	Yes	Magnetic resonance imaging showed potential retrolaminar involvement of the optic nerve and invasion of episclera
6	3.0	SCT, IVC, IAC	Progressive (PD)	No	No	
7	3.0	SCT, IAC	Progressive (PD)	No	No	
8	7.2	Naïve	Progressive (N/A)	No	No	
10	2.3	SCT, IAC	Progression‐free (PR)	No	No	
11	35.3	IAC	Progressive (PD)	No	Yes	Refractory to treatment
12	10.9	SCT, IAC	Progression‐free (PR)	No	No	
13	12.5	IAC	Progressive (PD)	No	No	
14	24.2	SCT, IAC	Progressive (PD)	No	No	
15	19.4	IAC	Progression‐free (PR)	No	No	
16	12.3	SCT, IVC	Progressive (PD)	Yes	Yes	Refractory to treatment
17	12.3	SCT	Progression‐free (SD)	No	No	
20	26.4	naïve	Progressive (N/A)	No	No	
21	10.3	IAC	Progression‐free (SD)	No	No	
23	4.0	SCT, IVC	Progression‐free (PR)	No	No	
24	6.2	SCT	Progression‐free (PR)	No	No	
25	31.0	IAC	Progression‐free (SD)	No	No	
26	1.5	SCT, IVC, IAC	Progressive (PD)	No	No	
36	3.0	SCT, IVC, IAC	Progression‐free (SD)	No	No	
38	4.4	SCT, IVC, IAC	Progressive (PD)	Yes	Yes	Refractory to treatment
39	6.2	SCT, IAC	Progression‐free (SD)	No	No	

IAC, intra‐arterial chemotherapy; IVC, intravitreal chemotherapy; N/A, not applicable (diagnostic samples); SCT, systemic chemotherapy.

The concentration of cfDNA was higher in the AH of retinoblastoma patients (26.1 ng; range 5.3–791) than in Controls (12.5 ng; range 3.6–108) (*p* = 0.0033) (supplementary material, Figure S1A). Upon stratification of retinoblastoma samples according to the progression status, the concentration of cfDNA in the AH obtained from patients in the Progressive RB group was similar to that in AH from patients included in the Progression‐free group (supplementary material, Figure S1B). We obtained sufficient cfDNA concentration to perform ddPCR assays for all samples.

### Analysis of nuclear and mitochondrial DNA sequences in AH for the detection of retinoblastoma in progression

**Figure 2:**
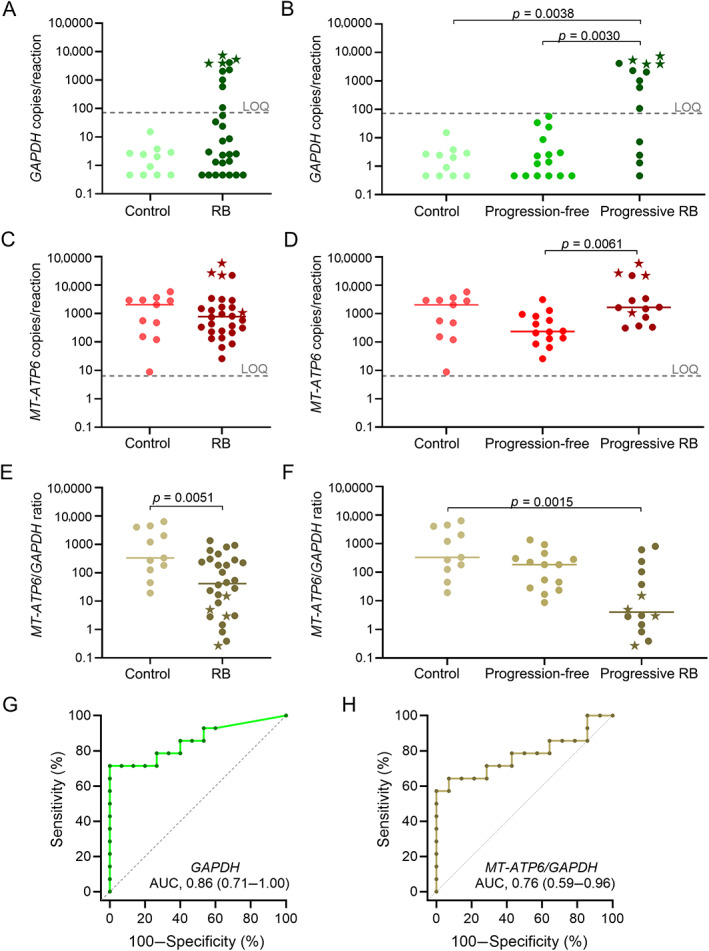
Analysis of nuclear DNA (*GAPDH*), mitochondrial DNA (*MT‐ATP6*), and mitochondrial‐to‐nuclear DNA ratios in AH (20 μl/sample) of Controls and RB patients. (A) Copies of *GAPDH* in AH of Controls and RB patients. AH samples in which *GAPDH* copies were below LOD are represented in the plot as 0.46, which is the lowest experimental value different from 0, divided by 2. Represented data are individual values (dots) and medians (lines). Dashed lines: LOQ. Data presented with stars correspond to AH samples with anterior chamber infiltration. (B) Copies of *GAPDH* in AH of Controls and RB patients classified as Progression‐free or Progressive RB. (C) Copies of *MT‐ATP6* in AH of Controls and RB patients. (D) Copies of *MT‐ATP6* in AH of Controls and RB patients classified as Progression‐free or Progressive RB. (E) Mitochondrial‐to‐nuclear DNA ratios in Controls and RB patients. (F) Mitochondrial‐to‐nuclear DNA ratios in Controls and RB patients classified as Progression‐free or Progressive RB. (G) ROC curve of *GAPDH* copies in AH to distinguish RB patients classified as Progression‐free or Progressive RB. AUC (95% CI). (H) ROC curve of mitochondrial‐to‐nuclear DNA ratios in AH to distinguish RB patients classified as Progression‐free or Progressive RB.

We quantified the copies of the conserved nuclear genes *GAPDH* and *B4GALNT1* and the mitochondrial gene *MT‐ATP6* in the AH samples. *GAPDH* copies were below the LOQ (72 copies per reaction) in all AH from Controls, with no detectable differences among conditions of these patients (supplementary material, Figure S2A). Thirty‐five percent of AH from retinoblastoma patients contained quantifiable copies (Figure 2A). All AH with quantifiable *GAPDH* belonged to patients in the Progressive RB group (Figure 2B). Among such patients, those with active tumors infiltrating the anterior chamber (patients 1, 2, 16, and 38; supplementary material, Figure S3) presented abundant *GAPDH* in their AH (4626 copies per reaction; range 3879–7438). Quantification of the second conserved nuclear DNA sequence, *B4GALNT1*, provided similar results (supplementary material, Figures S2B and S4A,B). *MT‐ATP6* copies were equally abundant in AH from Controls (2065 copies per reaction; range 9–5822) and in AH from retinoblastoma patients (777 copies per reaction; range 26–58,489) (*p* = 0.7236; Figure 2C). Counts of *MT‐ATP6* copies in the AH from patients in the Progression‐free group were significantly lower than those in the AH from patients in the Progressive RB group (Figure 2D). Because of the high number of *MT‐ATP6* copies and the low number of nuclear DNA sequence copies in Controls, the mitochondrial‐to‐nuclear DNA ratios in AH from Controls were significantly higher than those in AH from retinoblastoma patients (Figure 2E). Upon grouping retinoblastomas, the mitochondrial‐to‐nuclear DNA ratios in AH from Controls and from patients in the Progression‐free group were similar (Figure 2F). In contrast, AH from patients in the Progressive RB group had significantly lower mitochondrial‐to‐nuclear DNA ratios (Figure 2F). Quantification of the ratio with the *B4GALNT1* sequence provided similar results to *GAPDH* (supplementary material, Figure S4C,D).

Among retinoblastoma patients in the Progression‐free group, the area under the ROC curve (AUC) for *GAPDH* copies in AH was 0.86 (95% CI, 0.71–1.00), compared with the Progressive RB group. Optimal cut‐off value was 108 *GAPDH* copies per reaction, with a sensitivity of 71% and 100% specificity (Figure 2G). We obtained similar sensitivity and specificity using *B4GALNT1*, with an AUC of 0.81 (95% CI, 0.64–0.98) (supplementary material, Figure S4E). The sensitivity and specificity using the mitochondrial‐to‐nuclear ratio based on *GAPDH* copies were 64 and 93%, respectively (Figure 2H). We obtained similar results for the mitochondrial‐to‐nuclear ratio based on *B4GALNT1* copies (supplementary material, Figure S4F). We performed similar analyses eliminating data from patients with retinoblastoma infiltration in the anterior chamber, whose AH could be considered contaminated with cell DNA. We found that the cut‐off values did not change. Thus, we decided to leave these samples included in the whole study.

### Analysis of nuclear DNA in AH from enucleated eyes with available fundoscopy and histopathologic analyses

**Figure 3:**
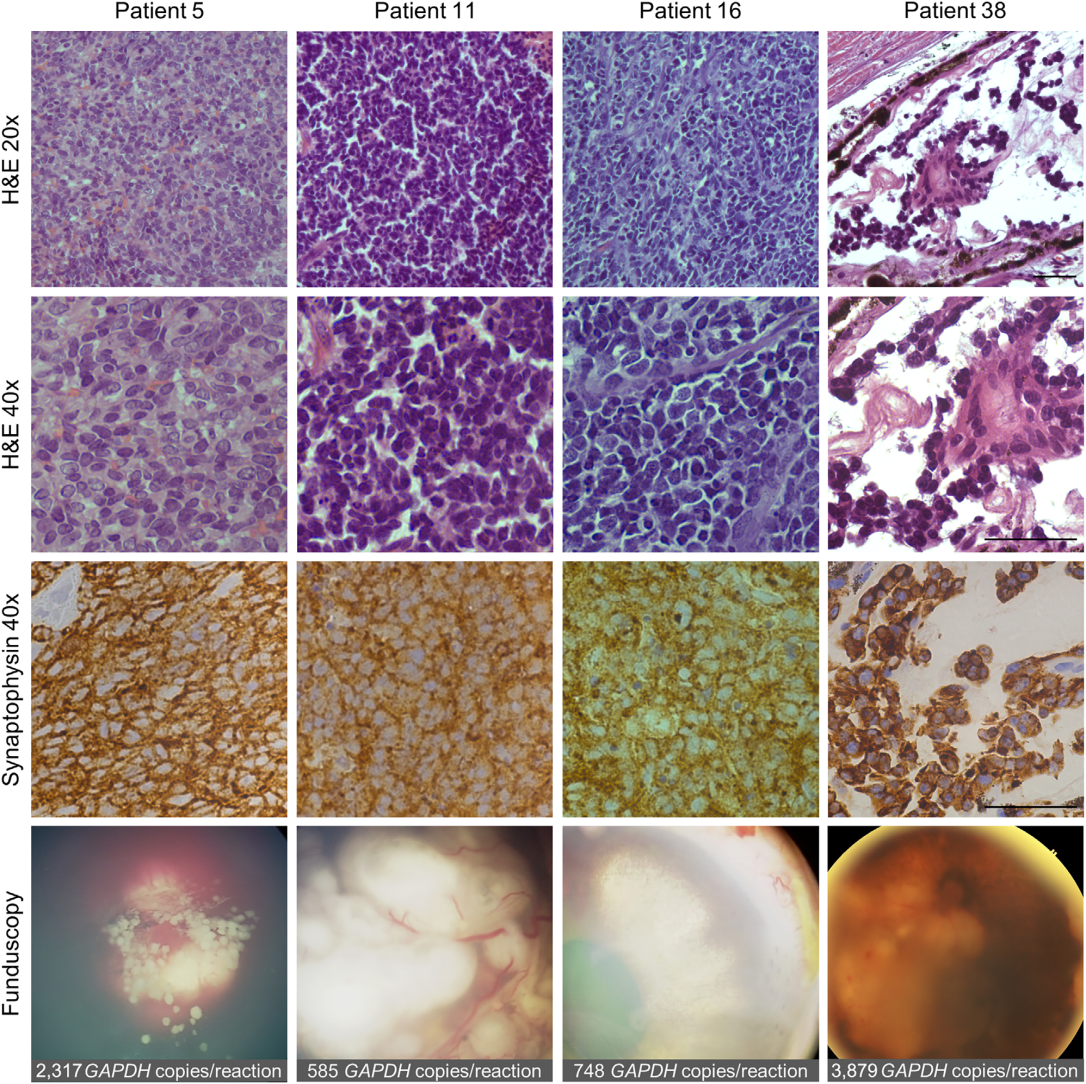
Pathology (H&E and synaptophysin), fundoscopy, and number of *GAPDH* copies per reaction in AH withdrawn at the time of enucleation, of four eyes containing large chemo‐resistant tumors in progression. Bars, 40 μm. Fundoscopy image of patient 5 was obtained on the day of enucleation. Images of patients 11, 16, and 38 were obtained 9, 14, and 16 days before enucleation, respectively.

We analyzed eyes from patients 5, 11, 16, and 38, enucleated due to tumor progression. Histology and fundoscopy images showed that the eyes of patients 5 and 16 contained large viable retinal tumors and profuse vitreous seeding, while the eye of patient 11 showed a large viable tumor with no seeding. Patient 38 showed a tumor in regression in the posterior segment and profuse vitreous seeding toward the anterior segment (Figure 3). Pathology analyses did not find postlaminar optic nerve invasion in any patient. Patients 5, 11, and 16 had prelaminar optic nerve involvement. We found choroidal invasion in the four enucleated eyes. Trans‐scleral involvement was present in patients 5 and 16. The number of *GAPDH* copies per reaction obtained from AH withdrawn before enucleations were in the range 585–3879, all above the cut‐off point (Figure 3).

### Follow‐up of tumor response to intravitreal chemotherapy through the analysis of *GAPDH* copies in AH

**Figure 4:**
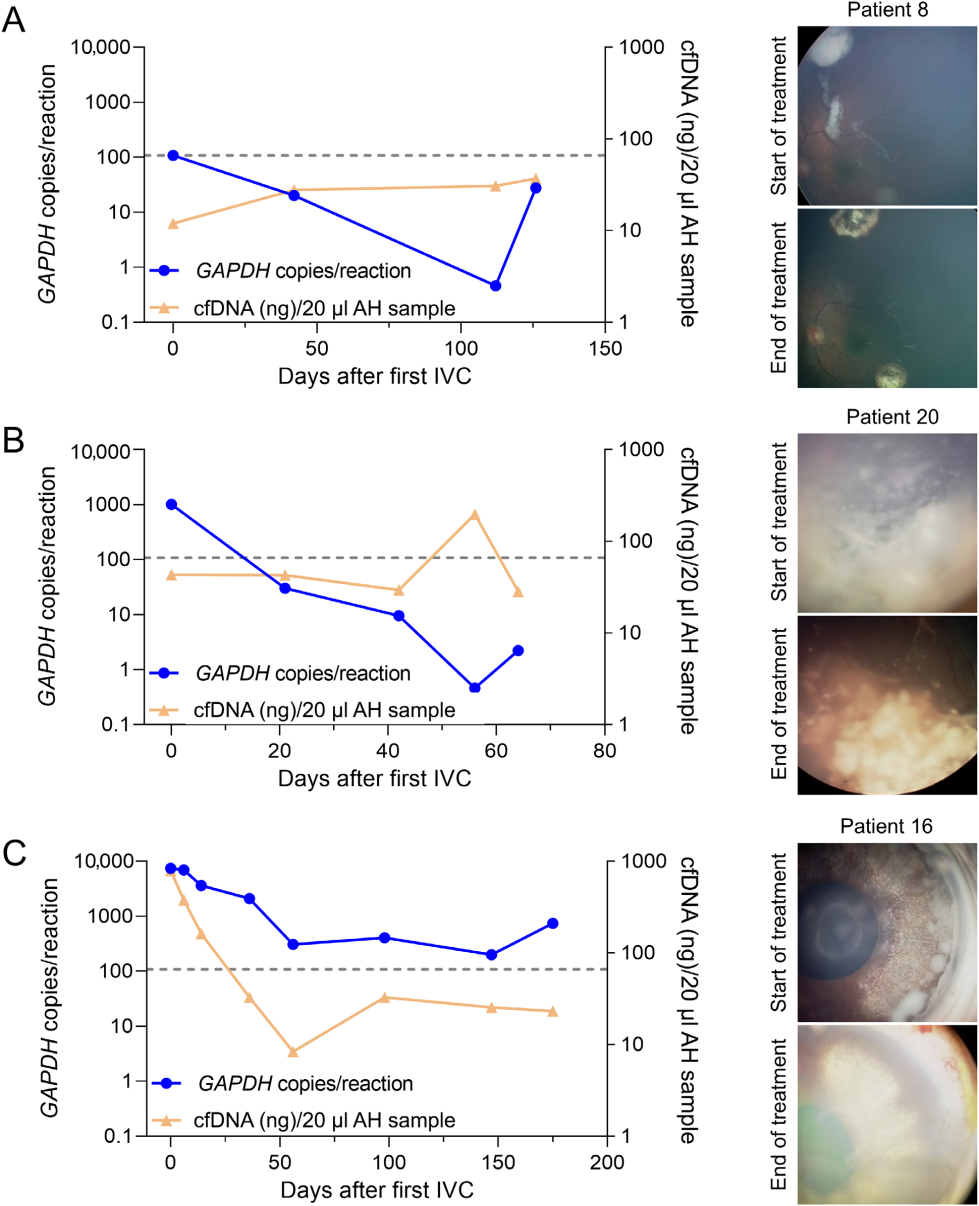
Analysis of *GAPDH* copies and cfDNA concentration in serial AH obtained during intravitreal chemotherapy of (A) patient 8, (B) patient 20, and (C) patient 16, and corresponding fundoscopy images at the start and end of treatment. Day 0 represents the sampling time of the first AH sample of the series. Dots are AH data and lines link serial data for each patient. Dashed lines: cut‐off point.

We obtained serial AH from patients 8, 16, and 20, treated with intravitreal chemotherapy and followed‐up by the standard of care fundoscopy method (RetCam). Patients 8 and 20 responded well to treatment. We obtained their first serial AH just before the administration of the first intravitreal dose (30 μg melphalan for patient 8 and 30 μg topotecan for patient 20). *GAPDH* copies in such first serial AH samples were above the cut‐off point and they decreased below the cut‐off point in the second serial AH, obtained before the second intravitreal dose (Figure 4A,B). The concentration of cfDNA in both patients remained constant during treatment (Figure 4A,B), likely because the mitochondrial copies predominated over nuclear copies (supplementary material, Figure S5). Fundoscopy showed that retinal tumors inactivated and became calcified, and the number of vitreous seeds diminished and calcified during the subsequent intravitreal doses (Figure 4A,B). Both patients remain in remission 623 and 339 days after the first intravitreal treatment.

Patient 16 did not respond well to treatment. She presented anterior chamber infiltration and had received 17 intravitreal melphalan doses before the collection of the first AH in our series. She received 30 μg topotecan doses during our study. Her initial number of *GAPDH* copies per reaction was 100 times above the cut‐off point and remained above in all the samples of the series (Figure 4C). The concentration of cfDNA decreased coincidently with the resolution of the anterior chamber infiltration upon receiving six intracameral doses of topotecan. Serial fundoscopy revealed a slight reduction of the seeding, but not of the retinal tumor (Figure 4C). She was finally enucleated due to tumor progression. At the time of enucleation, the number of *GAPDH* copies per reaction remained 10 times above the cut‐off point (Figure 4C).

## Discussion

Our study developed a sensitive ddPCR technique to quantify the content of conserved mitochondrial and nuclear DNA sequences in liquid biopsies of the anterior chamber of eyes of patients with intraocular retinoblastoma. Quantification of nuclear *GAPDH* copies in AH may complement fundoscopy to detect active intraocular retinoblastoma. The technique differentiates progression‐free and progressive retinoblastomas with the establishment of an optimal cut‐off point. Our method detected changes in tumor burden in serial patient‐matched samples.

We propose that the quantification of *GAPDH* copies in AH may be used as a follow‐up method for patients undergoing intravitreal chemotherapy treatments. Such analysis could complement RECIST criteria to assess objective response to treatment, especially when fundoscopy imaging is not clear due to opacity of the vitreous or vitritis. Our observation that tumors in progression have higher *GAPDH* gene copies in the AH than progression‐free tumors could be explained by the reduction in the tumor fraction of the cfDNA in treatment‐responsive tumors [8, 21]. Such results are concordant with the previous observation of cancer cells releasing greater amounts of nuclear DNA compared with non‐cancer cells [11, 12]. Two previous studies by the group at Children's Hospital Los Angeles reported the analysis of serial AH samples to follow‐up intraocular retinoblastoma by the calculation of the tumor fraction [8, 21]. They performed deep analysis of somatic copy number alterations in cfDNA and calculated the amplitude of the alterations at specific loci, such as gains of chromosome regions 1q, 2p, and 6p, and losses of 13q and 16q. The method is limited by the significant technical challenge posed to the clinical routine, and by the lack of copy number alterations suitable for follow‐up in at least 30% patients [8]. Methods using only one sensitive and reproducible technique, applicable to most patients, and easily interpretable, such as the one we propose here, add novel input to the managing of patients during routine treatments involving AH sampling. We acknowledge that we did not analyze copy number alterations in our patient cohort, and this is a limitation of our study. However, we considered unlikely that chromosome 12 is altered in retinoblastoma, according to recent published data [4].

Our method was not designed for the diagnosis of retinoblastoma. Nevertheless, we suggest that ddPCR for conserved nuclear sequences in AH could complement standard‐of‐care diagnosis through fundoscopy. Novel methods relying on patient‐specific analysis of genomic alterations are still not used in clinical practice. One example of such patient‐specific method is the detection of somatic *RB1* mutations in the cfDNA of the AH [7]. *RB1* is the most recurrently mutated gene in intraocular retinoblastoma [4] and there are at least 51 described types of *RB1* somatic mutations in this disease [22]. The *RB1* sequencing method would not detect 100% of retinoblastomas, because around 1% do not harbor *RB1* mutations [4]. A recent development of the French cooperative group presented a next‐generation sequencing method putting together a gene panel detecting *RB1* point variants, large genome rearrangements, and loss of heterozygosity in cfDNA from blood and AH [23]. Because deep sequencing is not always readily available in less specialized health centers, quantification of nuclear DNA in AH could be an alternative method. As a limitation, our study did not include enough number of AH samples from newly diagnosed patients, which impeded the calculation of the positive predictive value of the technique. Another limitation of our study is that we included few AH samples from patients with suitable differential diagnosis, such as Coats' disease, medulloepithelioma, and uveal melanoma. However, uveal melanoma virtually does not occur in children [24].

The AH is confined to one small compartment of the eye, thus it is likely that it contained concentrated tumor DNA and thus our method of nuclear DNA quantification was especially suitable to detect subtle changes in tumor content. Whether this method is adequate for liquid biopsies obtained from less compartmentalized tumors warrants further investigation.

## Author contributions statement

MC‐V and AMC conceived the work. All authors were involved in data collection and manuscript editing. MC‐V analyzed patient samples. MC‐V, SP‐J and AMC analyzed data. MC‐V generated the figures. AMC acquired funding. CL, JM and AMC provided resources. MC‐V and AMC wrote the original draft. AMC supervised the project.

## Supporting information


**Figure S1.** Quantification of cfDNA in AH (20 μl/sample) of Controls and retinoblastoma (RB) patients
**Figure S2.** Analysis of nuclear genes in AH (20 μl/sample) of Controls stratified upon eye conditions
**Figure S3.** Infiltration of the anterior chamber by retinoblastoma cells
**Figure S4.** Analysis of nuclear DNA (*B4GALNT1*), mitochondrial DNA (*MT‐ATP6*), and mitochondrial‐to‐nuclear DNA ratios in AH (20 μl/sample) of Controls and retinoblastoma (RB) patients
**Figure S5.** Analysis of *MT‐ATP6* copies in serial AH obtained during intravitreal chemotherapy
**Table S1.** Proportion of cell‐free DNA (cfDNA) and genomic DNA obtained in supernatant and cell pellets of primary retinoblastoma cells in culture
**Table S2.** Clinical details of control patients

## Data Availability

Data are available from the corresponding author on reasonable request.

## References

[1] Munier FL , Beck‐Popovic M , Chantada GL , *et al*. Conservative management of retinoblastoma: challenging orthodoxy without compromising the state of metastatic grace. “Alive, with good vision and no comorbidity”. Prog Retin Eye Res 2019; 73: 100764. 31173880 10.1016/j.preteyeres.2019.05.005

[2] Munier FL , Gaillard MC , Balmer A , *et al*. Intravitreal chemotherapy for vitreous disease in retinoblastoma revisited: from prohibition to conditional indications. Br J Ophthalmol 2012; 96: 1078–1083. 22694968 10.1136/bjophthalmol-2011-301450

[3] Berry JL , Xu L , Murphree AL , *et al*. Potential of aqueous humor as a surrogate tumor biopsy for retinoblastoma. JAMA Ophthalmol 2017; 135: 1221–1230. 29049475 10.1001/jamaophthalmol.2017.4097 PMC5710399

[4] Liu J , Ottaviani D , Sefta M , *et al*. A high‐risk retinoblastoma subtype with stemness features, dedifferentiated cone states and neuronal/ganglion cell gene expression. Nat Commun 2021; 12: 5578. 34552068 10.1038/s41467-021-25792-0 PMC8458383

[5] Zugbi S , Ganiewich D , Bhattacharyya A , *et al*. Clinical, genomic, and pharmacological study of MYCN‐amplified RB1 wild‐type metastatic retinoblastoma. Cancers (Basel) 2020; 12: 2714. 32971811 10.3390/cancers12092714 PMC7565107

[6] Xu L , Polski A , Prabakar RK , *et al*. Chromosome 6p amplification in aqueous humor cell‐free DNA is a prognostic biomarker for retinoblastoma ocular survival. Mol Cancer Res 2020; 18: 1166–1175. 32434859 10.1158/1541-7786.MCR-19-1262 PMC7415535

[7] Gerrish A , Stone E , Clokie S , *et al*. Non‐invasive diagnosis of retinoblastoma using cell‐free DNA from aqueous humour. Br J Ophthalmol 2019; 103: 721–724. 30745306 10.1136/bjophthalmol-2018-313005 PMC6709774

[8] Polski A , Xu L , Prabakar RK , *et al*. Cell‐free DNA tumor fraction in the aqueous humor is associated with therapeutic response in retinoblastoma patients. Transl Vis Sci Technol 2020; 9: 30. 10.1167/tvst.9.10.30 PMC7533735 33062393

[9] Abramson DH , Mandelker D , Francis JH , *et al*. Retrospective evaluation of somatic alterations in cell‐free DNA from blood in retinoblastoma. Ophthalmol Sci 2021; 1: 100015. 36246006 10.1016/j.xops.2021.100015 PMC9560572

[10] Fazzini F , Schöpf B , Blatzer M , *et al*. Plasmid‐normalized quantification of relative mitochondrial DNA copy number. Sci Rep 2018; 8: 15347. 30337569 10.1038/s41598-018-33684-5 PMC6194030

[11] Perdas E , Stawski R , Kaczka K , *et al*. Altered levels of circulating nuclear and mitochondrial DNA in patients with papillary thyroid cancer. Sci Rep 2019; 9: 14438. 31594998 10.1038/s41598-019-51000-7 PMC6783406

[12] Haupts A , Vogel A , Foersch S , *et al*. Comparative analysis of nuclear and mitochondrial DNA from tissue and liquid biopsies of colorectal cancer patients. Sci Rep 2021; 11: 16745. 34408162 10.1038/s41598-021-95006-6 PMC8373949

[13] Triska P , Kaneva K , Merkurjev D , *et al*. Landscape of germline and somatic mitochondrial DNA mutations in pediatric malignancies. Cancer Res 2019; 79: 1318–1330. 30709931 10.1158/0008-5472.CAN-18-2220 PMC6445760

[14] Portoukalian J , David MJ , Gain P , *et al*. Shedding of GD2 ganglioside in patients with retinoblastoma. Int J Cancer 1993; 53: 948–951. 8473052 10.1002/ijc.2910530614

[15] Pascual‐Pasto G , Olaciregui NG , Vila‐Ubach M , *et al*. Preclinical platform of retinoblastoma xenografts recapitulating human disease and molecular markers of dissemination. Cancer Lett 2016; 380: 10–19. 27319373 10.1016/j.canlet.2016.06.012

[16] Pascual‐Pasto G , Bazan‐Peregrino M , Olaciregui NG , *et al*. Therapeutic targeting of the RB1 pathway in retinoblastoma with the oncolytic adenovirus VCN‐01. Sci Transl Med 2019; 11: eaat9321. 30674657 10.1126/scitranslmed.aat9321

[17] Cuadrado‐Vilanova M , Liu J , Paco S , *et al*. Identification of immunosuppressive factors in retinoblastoma cell secretomes and aqueous humor from patients. J Pathol 2022; 257: 327–339. 35254670 10.1002/path.5893

[18] Berry JL , Munier FL , Gallie BL , *et al*. Response criteria for intraocular retinoblastoma: RB‐RECIST. Pediatr Blood Cancer 2021; 68: e28964. 33624399 10.1002/pbc.28964 PMC8049511

[19] Skaltsa K , Jover L , Carrasco JL . Estimation of the diagnostic threshold accounting for decision costs and sampling uncertainty. Biom J 2010; 52: 676–697. 20976697 10.1002/bimj.200900294

[20] Youden WJ . Index for rating diagnostic tests. Cancer 1950; 3: 32–35. 15405679 10.1002/1097-0142(1950)3:1<32::aid-cncr2820030106>3.0.co;2-3

[21] Xu L , Kim ME , Polski A , *et al*. Establishing the clinical utility of ctDNA analysis for diagnosis, prognosis, and treatment monitoring of retinoblastoma: the aqueous humor liquid biopsy. Cancers (Basel) 2021; 13: 1282. 33805776 10.3390/cancers13061282 PMC8001323

[22] Kooi IE , Mol BM , Massink MPG , *et al*. Somatic genomic alterations in retinoblastoma beyond RB1 are rare and limited to copy number changes. Sci Rep 2016; 6: 25264. 27126562 10.1038/srep25264 PMC4850475

[23] Le Gall J , Dehainault C , Benoist C , *et al*. Highly sensitive detection method of retinoblastoma genetic predisposition and biomarkers. J Mol Diagn 2021; 23: 1714–1721. 34656762 10.1016/j.jmoldx.2021.08.014

[24] Singh AD , Turell ME , Topham AK . Uveal melanoma: trends in incidence, treatment, and survival. Ophthalmology 2011; 118: 1881–1885. 21704381 10.1016/j.ophtha.2011.01.040

