# Supplementary material for: Follow‐up of intraocular retinoblastoma through the quantitative analysis of conserved nuclear DNA sequences in aqueous humor from patients
Source: J Pathol Clin Res. 2022 Sep 23;9(1):32–43. doi: 10.1002/cjp2.296 (PMC9732679; doi:10.1002/cjp2.296)
Supplement: Supplementary file 1 — Figure S1. Quantification of cfDNA in AH (20 μl/sample) of Controls and retinoblastoma (RB) patients Figure S2. Analysis of nuclear genes in AH (20 μl/sample) of Controls stratified upon eye conditions Figure S3. Infiltration of the anterior chamber by retinoblastoma cells Figure S4. Analysis of nuclear DNA (B4GALNT1), mitochondrial DNA (MT‐ATP6), and mitochondrial‐to‐nuclear DNA ratios in AH (20 μl/sample) of Controls and retinoblastoma (RB) patients Figure S5. Analysis of MT‐ATP6 copies in serial AH obtained during intravitreal chemotherapy Table S1. Proportion of cell‐free DNA (cfDNA) and genomic DNA obtained in supernatant and cell pellets of primary retinoblastoma cells in culture Table S2. Clinical details of control patients [file CJP2-9-32-s001.pdf]

Follow-up of intraocular retinoblastoma through the quantitative analysis of conserved nuclear DNA sequences in aqueous humor from patients

M Cuadrado-Vilanova *et al.* *J Pathol Clin Res,* https://doi.org/10.1002/cjp2.296

Supplementary Figures S1–S5

Supplementary Tables S1,S2

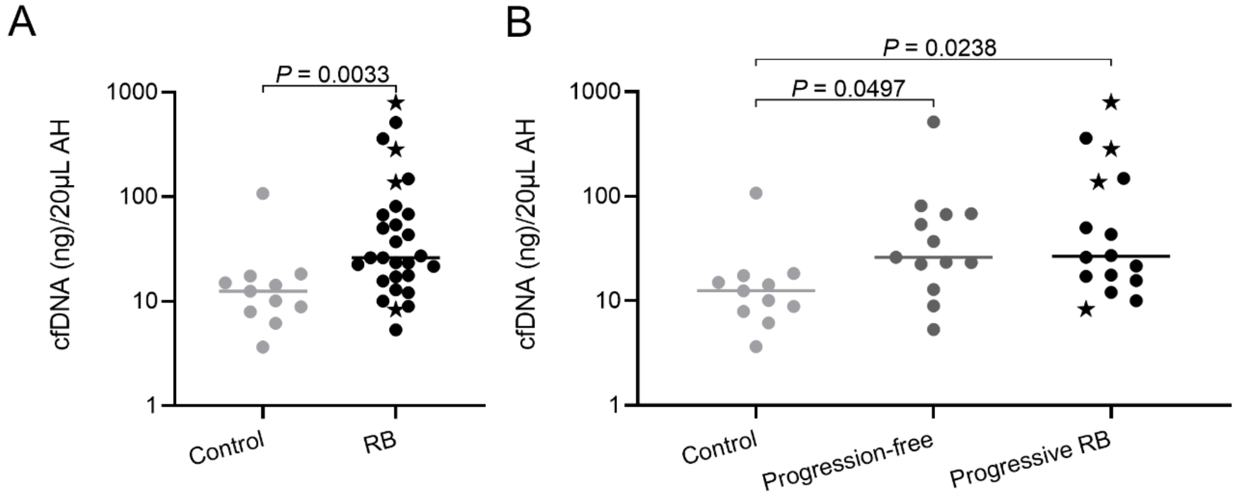

Figure S1. Quantification of cfDNA in AH (20 µl/sample) of controls and retinoblastoma (RB) patients. (A) cfDNA concentration in AH of control and RB patients. (B) cfDNA concentration in AH of Controls and RB patients classified as Progression-free or Progressive RB. Represented data are individual values (dots) and medians (lines). Data presented with stars correspond to samples with anterior chamber infiltration.

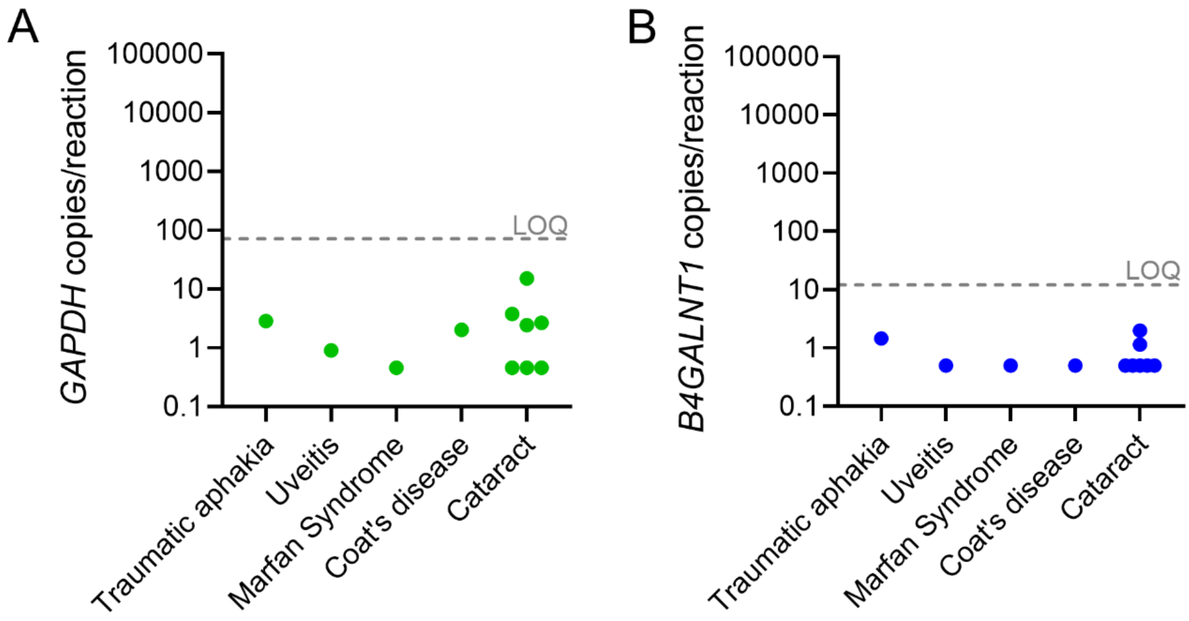

Figure S2. Analysis of nuclear genes in AH (20 µl/sample) of Controls stratified upon eye conditions. (A) Copies of *GAPDH* in AH. AH samples in which GAPDH copies were below LOD are represented in the plot as 0.46, which is the lowest experimental value different from 0, divided by 2. Presented data are individual values (dots). (B) Copies of *B4GALNT1* in AH. AH samples in which *B4GALNT1* copies were below LOD are represented in the plot as 0.5, which is the lowest experimental value different from 0, divided by 2. Presented data are individual values (dots).

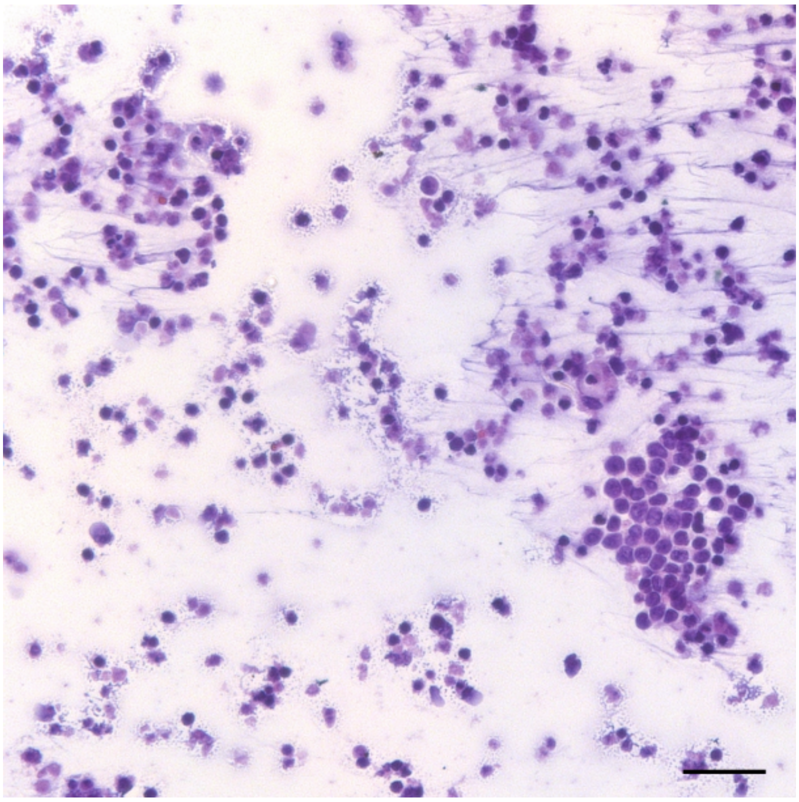

Figure S3. Infiltration of the anterior chamber by retinoblastoma cells. This image shows hematoxylin and eosin staining of the AH sample of patient 2. Bar, 40 µm.

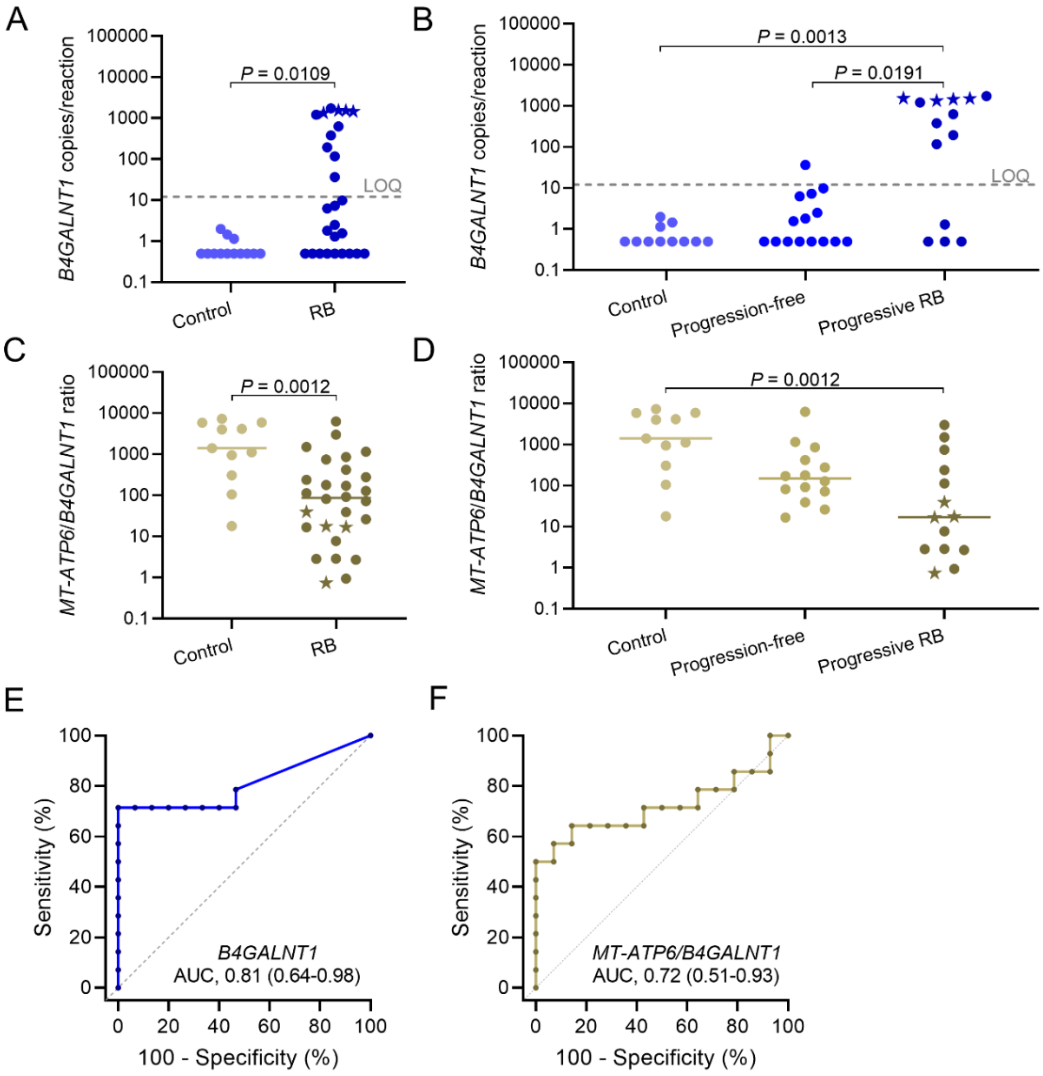

Figure S4. Analysis of nuclear DNA (*B4GALNT1*), mitochondrial DNA (*MT-ATP6*), and mitochondrial-to-nuclear DNA ratios in AH (20 µl/sample) of Controls and retinoblastoma (RB) patients. (A) Copies of *B4GALNT1* in AH of Controls and RB patients. AH samples in which *B4GALNT1* copies were below LOD are represented in the plot as 0.5, which is the lowest experimental value different from 0, divided by 2. Data represented with stars correspond to samples with anterior chamber infiltration. (B) Copies of *B4GALNT1* in AH of Control and patients classified as Progression-free or Progressive RB. (C) Mitochondrial-to-nuclear DNA ratios in Controls and RB patients. (D) Mitochondrial-to-nuclear DNA ratios in Controls and patients classified as Progression-free or Progressive RB. (E) Receiver-operating-characteristic (ROC) curve of *B4GALNT1* copies in AH to distinguish RB patients classified as Progression-free or Progressive RB. AUC: area under the ROC curve (95% confidence interval). (F) ROC curve of mitochondrial-to-nuclear DNA ratios in AH to distinguish RB patients classified as Progression-free or Progressive RB.

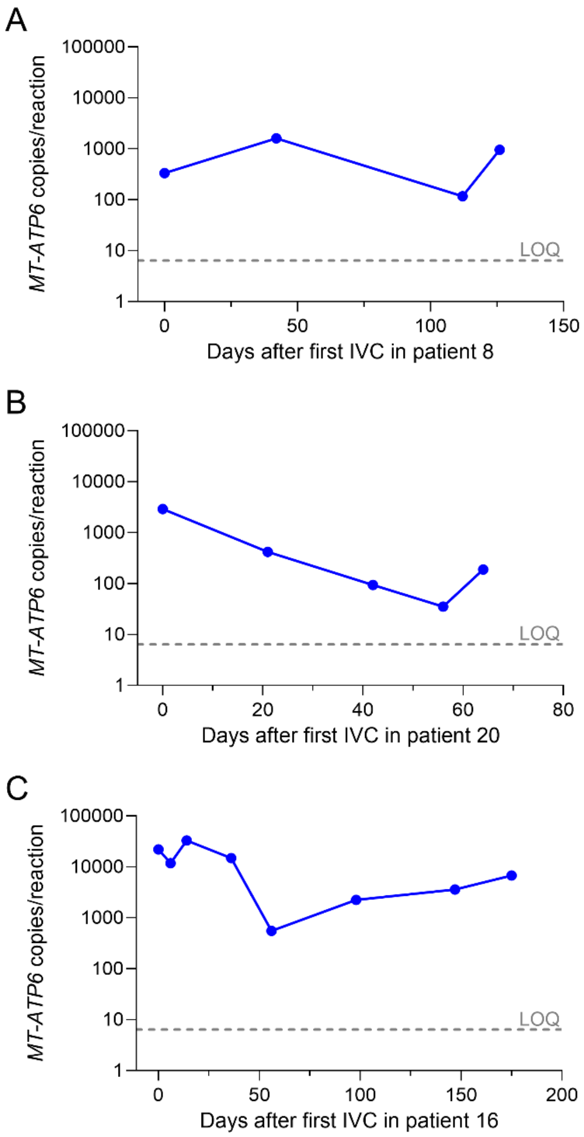

Figure S5. Analysis of *MT-ATP6* copies in serial AH obtained during intravitreal chemotherapy. (A) patient 8, (B) patient 20 and (C) patient 16. Day 0 represents the collection time of the first AH sample of the series. Dots are AH data and lines link serial data for each patient. Dashed lines: LOQ.

Supplementary Tables S1,S2

Table S1. Proportion of cell-free DNA (cfDNA) and genomic DNA obtained in supernatant and cell pellets of primary retinoblastoma cells in culture.

Cell culture identification	DNA fraction	DNA concentration (ng)/ 100,000 cells	cfDNA / genomic DNA (%)
HSJD-RBVS-1	genomic DNA	1033.60	
	cfDNA	45.24	4.38
HSJD-RBT-5	genomic DNA	7760.00	
	cfDNA	171.42	2.21
HSJD-RBT-7	genomic DNA	2972.80	
	cfDNA	20.78	0.70
HSJD-RBVS-10	genomic DNA	2886.20	
	cfDNA	127.31	4.41
HSJD-RBT-14	genomic DNA	3609.54	
	cfDNA	69.89	1.94
HSJD-RBVS-29	genomic DNA	1704.51	
	cfDNA	63.28	3.71
HPG-RBG-396	genomic DNA	3257.16	
	cfDNA	26.44	0.81
HPG-RBT-12L	genomic DNA	747.41	
	cfDNA	47.22	6.32

Table S2. Clinical details of control patients

Patient ID	Age (years)	Pathology
27	14.0	Uveitis
28	13.5	Marfan syndrome
29	6.6	Coats' disease
30	7.7	Traumatic aphakia
31	58.8	Cataract
32	67.3	Cataract
33	54.3	Cataract
34	77.4	Cataract
35	77.4	Cataract
40	16.4	Cataract
41	0.3	Cataract
